# Characteristics and related factors of family functioning in Chinese families during early pregnancy

**DOI:** 10.3389/fpsyg.2023.1102796

**Published:** 2023-02-15

**Authors:** Xuemei Qin, Weiling Zhang, Shuyin Xu, Mohan Ma, Xing Fan, Xueqing Nie, Jin Liu, Yumeng Ju, Li Zhang, Lingjiang Li, HaoLun Li, Bangshan Liu, Yan Zhang

**Affiliations:** ^1^Department of Psychiatry, and National Clinical Research Center for Mental Disorders, The Second Xiangya Hospital of Central South University, Changsha, Hunan, China; ^2^Mental Health Institute of Central South University, China National Technology Institute on Mental Disorders, Hunan Technology Institute of Psychiatry, Hunan Key Laboratory of Psychiatry and Mental Health, Hunan Medical Center for Mental Health, Changsha, China; ^3^Changsha Hospital for Maternal and Child Health Care, Changsha, Hunan, China

**Keywords:** family functioning, females and partners, early pregnancy, mood symptoms, related factors, characteristics, Behavior Control

## Abstract

**Introduction:**

Family functioning has been found to significantly impact each family member’s health mentally, physically, and socially. A number of the research has focused on the impact of impaired family functioning in general, but limited studies explore family functioning in the vulnerable period, early pregnancy. Therefore, the study aimed to investigate the characteristics and related factors in Chinese females and partners during early pregnancy.

**Methods:**

The cross-sectional study enrolled 226 pregnant women and 166 partners. Assessment tools included the McMaster Family Assessment Device (FAD), Edinburgh Postnatal Depression Scale, Patient Health Questionnaire-9, Generalized Anxiety Disorder 7-Item, Social Support Rating Scale, and Quality of Life Enjoyment and Satisfaction Questionnaire, Short Form. Correlation analysis was applied to investigate the related factors.

**Results:**

In the present study, FAD-Behavior Control (BC) was the only dysfunctional dimension and had the highest dysfunctional rates than other dimensions. Length of time living with a partner, depressive and anxious symptoms, and quality of life were all associated with the dysfunctional family functioning of BC.

**Conclusions:**

The study reinforced the important clues of family functioning during early pregnancy. Also, it provided new entry points for the general population and healthcare providers to minimize the negative impact that impaired family function might bring to a family.

## 1. Introduction

According to the McMaster Model of Family Functioning (MMFF) theory (Epstein et al., 1983), family functioning is defined as whether a family can perform various tasks for the family and its members’ healthy development. Family functioning considers the family as a system. It examines the overall function of the family system in seven dimensions, including Problem Solving (PS), Communication (CM), Roles (RL), Affective Responsiveness (AR), Affective Involvement (AI), Behavior Control (BC), and General Functioning (GF; Epstein et al., 1983). It is a fundamental indicator of family system functioning within the family system and is closely related to the individual’s mental health (Shek, 2002b). However, family functioning is more likely to involve more challenges during different periods of the family life cycle. The Modernized Family Life Circle theory separates the development of a family into five main stages, including young single, young married without children, other young, middle-aged, and older. Within these stages, 13 sub-categories indicate different turning points of family development, such as marriage, childbirth, divorce, or widowed (Murphy and Staples, 1979). These periods are considered more vulnerable during family development as a family is probably experiencing role change, responsibility shifting, and living environment changes.

Early pregnancy is also essential in the development of a family, which faces more challenges at this time. Of particular note is that contextual theory suggests that personal development and family functioning are viewed as adaptive processes to external challenges in biological, perceptual, cognitive, behavioral, and interpersonal perspectives (Luthar, 2003). Pregnant families certainly face more complex situations that require adjustment by each family member throughout these perspectives. They are more likely to be challenged by shifting responsibilities, changing roles, and psychosocial distress during this period. As previous studies suggested, expectant fathers must adapt to working and family responsibilities, adjust to a new role, and so on. These responsibilities would result in tiredness and stress, which increases irritability in facing problems (Fägerskiöld, 2008) and further impair family functioning possibly. Also, the expectant mother will be challenged by stress from occupational shifting (Horne et al., 2005), multiple role changes in life, as well as anxiety from various somatic discomfort, and psychological changes (Bjelica and Kapor-Stanulovic, 2004). Researchers and health professionals emphasize the importance of family functioning, and early pregnancy is considered a vulnerable time in the family, which reinforces the importance of paying more attention to family functioning in early pregnancy.

Generally, family functioning is found to influence people’s health mentally and socially and is associated with mood disorders. According to previous research, impaired family functioning is likely to increase the risk of depression and is associated with anxiety (Wang et al., 2013). Not only depression and anxiety but family functioning and bipolar disorder episodes mutually fluctuate and influence each other (Uebelacker et al., 2006). The impaired family functioning would also impact people socially. Researchers revealed that teenagers with an impaired family functioning environment are likelier to engage in violence and later violence perpetration (Gorman-Smith et al., 2004). Besides, family functioning also plays a vital role in the health of family members during pregnancy. On the one hand, impaired family functioning would increase the likelihood of symptoms of depression and anxiety in pregnant females and their partners during early pregnancy (Qin et al., 2022). On the other hand, maternal exposure to poor family functioning during pregnancy would influence the offspring’s brain development in the hippocampus (Xerxa et al., 2021) and increase their vulnerability to being victimized by peers in the future (Lereya and Wolke, 2013).

Furthermore, earlier studies have identified the importance of dimensions in family functioning. CM and GF of family functioning could directly predict anxiety in the general population (Ballash et al., 2006), and four dimensions in family functioning (PS, CM, RL, and GF) are specifically associated with suicide attempts in bipolar disorder (Berutti et al., 2016). Therefore, to contribute to the understanding of family functioning, the dimensions should be closely studied as they would play a role in updating the guidance of family education and interventions.

However, limited research has been conducted in early pregnancy groups. Thus, this study aimed to explore the characteristics of family functioning in early pregnancy among Chinese women and partners and to examine the associated factors simultaneously. Recognizing and promoting an understanding of the underlying characteristics of family functioning during pregnancy will facilitate the development of more effective family interventions for early pregnant families.

## 2. Materials and methods

### 2.1. Participants and procedure

Pregnant females and partners were recruited from the outpatient clinic from December 2020 to July 2021. Except for 37 females who were excluded based on the exclusion criteria and 20 females with incomplete information, the current study sample comprised 226 pregnant women in total. One hundred and sixty-six partners participated in the study, except the 25 partners excluded based on the exclusion criteria and 6 partners with incomplete information. Following the inclusion criteria, we included participants who were ethnic Han, right-handed, received junior high school education or above, and lived with a partner. For pregnant females, we only included those aged 18–40 who were pregnant within 13^+6^ weeks. Subjects with a current or any history of mental disorders (such as schizophrenia, major depressive disorder, anxiety disorder, bipolar disorders, and so on), psychoactive substance dependence, and organic brain disease or severe physical illness (such as severe infections, trauma, immune disorders, or other significant medical conditions) were excluded from this study. Each participant signed an informed consent form, and the ethics committee approved the study.

### 2.2. Assessments

#### 2.2.1. Family functioning

Compared to the other scales used to assess family functioning, such as the Family Intimacy and Adaptability Scale, Family Environment Scale, Family Adaptation, Partnership, Growth, Affection, Resolve Questionnaire, and so on, the McMaster Family Assessment Device (FAD) is widely used and allows for a more comprehensive assessment of family functioning. Therefore, in the current study, the FAD was used to assess pregnant females’ and male partners’ family functioning. The scale was developed by Epstein et al. in 1983 based on the MMFF theory (Epstein et al., 1983), which included PS, CM, RL, AR, AI, BC, and GF as subscales. It allows family members to self-assess whether the family is functioning well and identify possible family system problems. The scale has good reliability and validity (Miller et al., 2000), and its Chinese version has good psychometric properties (Shek, 2002a) and is also widely used in China.

Family assessment device-problem solving (FAD-PS) includes six items evaluating the family’s ability to resolve problems and effective functioning (items 2, 12, 24, 38, 50, and 60). FAD-CM focuses on the effectiveness of verbal information exchange within the family (items 3, 14, 18, 22, 29, 35, 43, 52, and 59), which is consistent with nine items that measure whether they understand each other’s concerns and talk about their emotions and thoughts directly. In addition, FAD-RL is defined as repetitive patterns of behavior in which individuals performed family functions (items 4, 8, 10, 15, 23, 30, 34, 40, 45, 53, and 58). FAD-AR evaluates family members’ responses to emotional stimuli with appropriate quality and quantity in feelings (items 9, 19, 28, 39, 49, and 57) and focuses on whether family members express their emotions in front of everyone and if the family’s intimate and attentive connection is essential. FAD-AI evaluates the extent to which families show interest in and value the activities and interests of family members (items 5, 13, 25, 33, 37, 42, and 54). FAD-BC dimension evaluates the behavior pattern from three perspectives: situations involved in physical danger, psychobiological needs, and social behavior (items 7, 17, 20, 27, 32, 44, 47, 48, and 55), precisely, the sub-scale asks people to assess whether or not they felt helpless when an accident happened, whether there is a rigorous principle that could not be disobeyed, and if family members had their regulations in life to obey. Lastly, FAD-GF focuses on 12 items (items 1, 6, 11, 16, 21, 26, 31, 36, 41, 46, 51, and 56), which includes whether family members support and trust each other and whether they have been supported to be who they are original and make decisions together in a harmonious environment.

The score for each subscale is equal to the average for each item, ranging from 1.0 to 4.0 points, with higher scores indicating poorer family functioning. The cut-off scores include FAD-PS > 2.20, FAD-CM > 2.20, FAD-RL > 2.30, FAD-AR > 2.20, FAD-AI>2.10, FAD-BC > 1.90, and FAD-GF > 2.00. In the present study, a higher subscale score than the cut-off score was defined as dysfunctional family functioning for that dimension (Tamplin and Gooyer, 2001; Wang et al., 2013).

#### 2.2.2. Anxious and depressive symptoms

Considering the potential relationship between family functioning and anxious and depressive symptoms based on previous research, the Edinburgh Postnatal Depression Scale (EPDS) and Patient Health Questionnaire-9 (PHQ-9) were used to evaluate pregnant females’ and male partners’ depressive symptoms, respectively. Generalized Anxiety Disorder-7 (GAD-7) was used to assess anxious symptoms. Those three scales are commonly used in clinical practice and show good reliability and validity.

#### 2.2.3. Psychosocial factors

Psychosocial factors such as social support and quality of life may be associated with family functioning according to previous research. In the present study, pregnant females have been assessed by the Social Support Rating Scale (SSRS) to measure self-reported social support (Xiao, 1994), and Quality of Life Enjoyment and Satisfaction Questionnaire, Short Form (Q-LES-Q-SF; Jean et al., 1993) to assess the quality of life. Both scales are relatively widely used in their respective domains and have been shown to have good psychometric properties.

### 2.3. Statistical analysis

The statistical analyses were performed on IBM SPSS for Windows, version 20.0 (IBM Corp., Armonk, NY, United States). Data are presented in mean (standard deviation, SD), median (interquartile range, IQR), or *n* (%) in different categories. One sample *t*-test, two independent samples *t*-test, Chi-square test, Mann–Whitney *U* test, Spearman correlation and partial correlation analysis were used to investigate participants’ family functioning characteristics and related factors. Statistical significance was set as a two-tailed *p* < 0.05.

## 3. Results

### 3.1. Demographic information and clinical characteristics of participants

As shown in Table 1, the mean age of the pregnant females was about 29 years old (29.46 ± 4.12). Most of them were married (85.4%) and had lived with their partners for an average of 3 years. Male participants were about 31 years old (31.28 ± 4.48), and their average length of education was 15 years (15.18 ± 2.52). There were 84.9% of partners married and 98.8% employed.

**Table 1:** Demographic information and clinical characteristics of participants.

Item	Pregnant females (*n* = 226)	Partners (*n* = 166)
Demographic information		
Age (years)	29.46 ± 4.12	31.28 ± 4.48
BMI (kg/m^2^)	21.46 ± 3.00	23.79 ± 3.39
Years of education	14.97 ± 2.35	15.18 ± 2.52
Marital status, married (%)	193 (85.4)	141 (84.9)
Length of time living with a partner (years)	3 (4)	2.5 (4)
Occupational status, in employment (%)	171 (75.7)	164 (98.8)
Clinical characteristics		
History of drinking (%)	12 (5.3)	90 (54.2)
History of smoking (%)	11 (4.9)	79 (47.6)
Planned pregnancy (%)	143 (63.3)	101 (60.8)
Gestational weeks	6.46 ± 1.76	6.45 ± 1.73
Total scores of EPDS	6 (5)	–
Total scores of PHQ-9	–	4 (6)
Total scores of GAD-7	3 (4)	2 (6)
Total scores of SSRS	38.05 ± 5.80	–
Total scores of Q-LES-Q-SF	53.06 ± 7.25	–

Data are shown as mean ± standard deviation, median (interquartile range), or *n* (%) in different categories.

BMI, Body Mass Index; EPDS, Edinburgh Postnatal Depression Scale; PHQ-9, Patient Health Questionnaire-9; GAD-7, Generalized Anxiety Disorder 7-item; SSRS, Social Support Rating Scale; Q-LES-Q-SF, Quality of Life Enjoyment and Satisfaction Questionnaire, Short Form.

In clinical characteristics, we found that 5.3% of females and 54.2% of males were involved in a drinking history, defined as consuming an alcoholic beverage at least once per month on average, 3 months before pregnancy. Meanwhile, 4.9% of females and 47.6% of males involved a smoking history, defined as smoking tobacco products at least once a day for a month or longer before pregnancy. More than half of females (63.3%) and male partners (60.8%) consider current pregnancy as planned. Additionally, median scores of EPDS, PHQ-9, and GAD-7 in participants were all less than 10 points.

### 3.2. Characteristics of family functioning in participants

**Figure 1:**
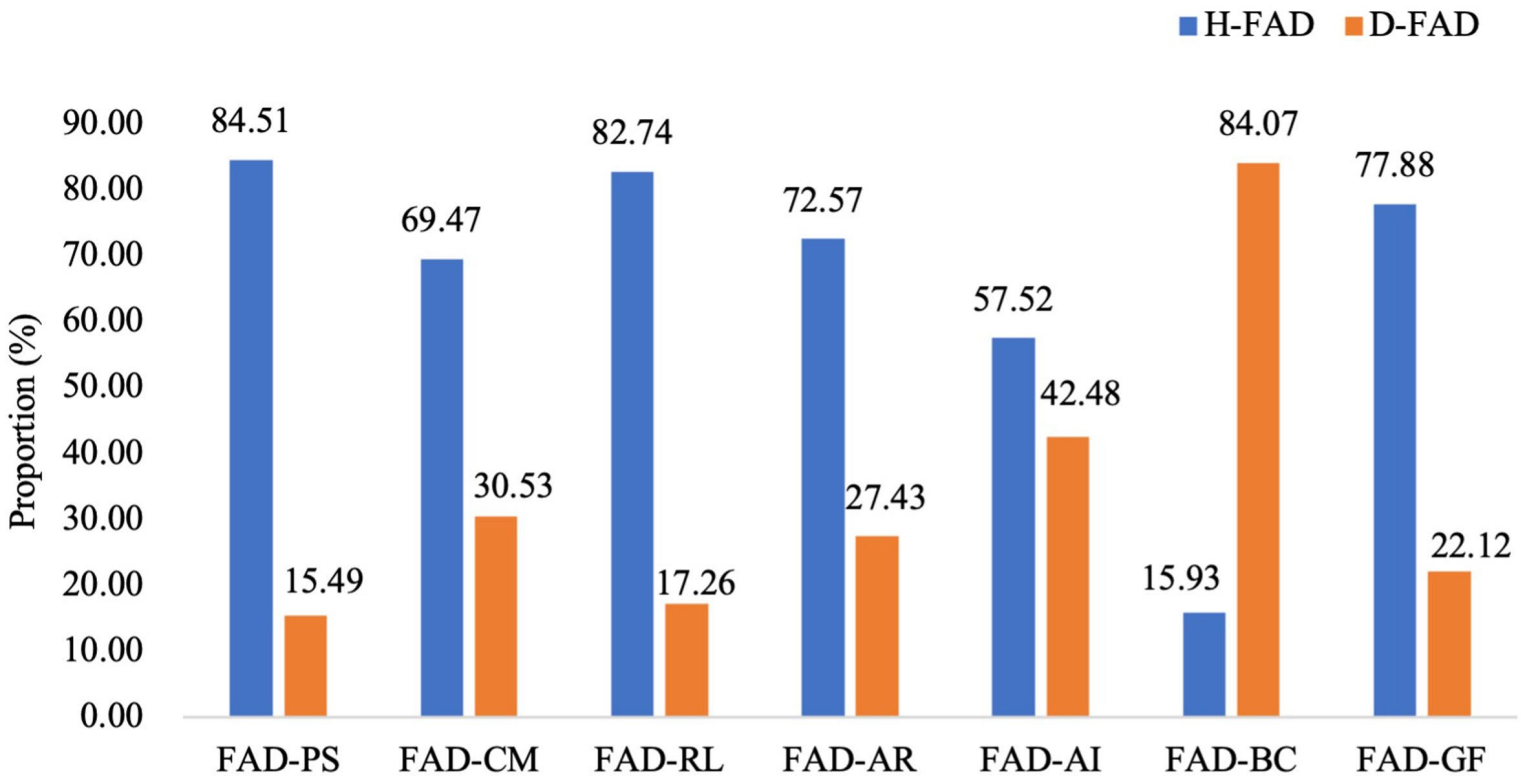
Proportion of pregnant females with healthy and dysfunctional Family Assessment Device (FAD) subscales (%).

In pregnant females, as shown in Table 2, mean scores of FAD-PS, CM, RL, AR, AI, and GF were below the cut-off scores (all *p* < 0.01). The mean scores of FAD-BC were higher than the cut-off scores (*t* = 17.249, *p* < 0.001). Meanwhile, we analyzed the proportion of pregnant females with healthy and dysfunctional FAD subscales, and data was presented in Figure 1. Comparing the proportion of dysfunctional FAD subscales to that of healthy subscales among females, the proportion of dysfunctional FAD-BC was the only one that prominently exceeded the proportion of the corresponding healthy subscale (84.07% versus 15.93%).

**Table 2:** Characteristics of family functioning in participants.

Item	Cut-off scores	Pregnant females (*n* = 226)			Partners (*n* = 166)		
		Mean ± SD	*t*	*P_1_* value^a^	Mean ± SD	*t*	*P_2_* value^a^
FAD-PS	2.20	1.89 ± 0.40	−11.448	**<0.001**	2.00 ± 0.39	−6.396	**<0.001**
FAD-CM	2.20	1.99 ± 0.43	−7.438	**<0.001**	2.11 ± 0.43	−2.673	**0.008**
FAD-RL	2.30	2.02 ± 0.36	−11.662	**<0.001**	2.16 ± 0.34	−5.327	**<0.001**
FAD-AR	2.20	2.01 ± 0.48	−5.888	**<0.001**	2.06 ± 0.42	−4.370	**<0.001**
FAD-AI	2.10	2.02 ± 0.44	−2.714	**0.007**	2.08 ± 0.48	−0.516	0.607
FAD-BC	1.90	2.21 ± 0.27	17.249	**<0.001**	2.19 ± 0.32	11.576	**<0.001**
FAD-GF	2.00	1.76 ± 0.44	−8.255	**<0.001**	1.84 ± 0.39	−5.214	**<0.001**

Bold values indicate statistical significance; ^a^One-Sample t-test. *P_1_* value: differences between subscale scores of FAD and cut-off scores in pregnant women; *P_2_* value: differences between subscale scores of FAD and cut-off scores in partners. SD, Standard Deviation; FAD, Family Assessment Device; PS, Problem Solving; CM, Communication; RL, Roles; AR, Affective Responsiveness; AI, Affective Involvement; BC, Behavior Control; GF, General Functioning.

**Figure 2:**
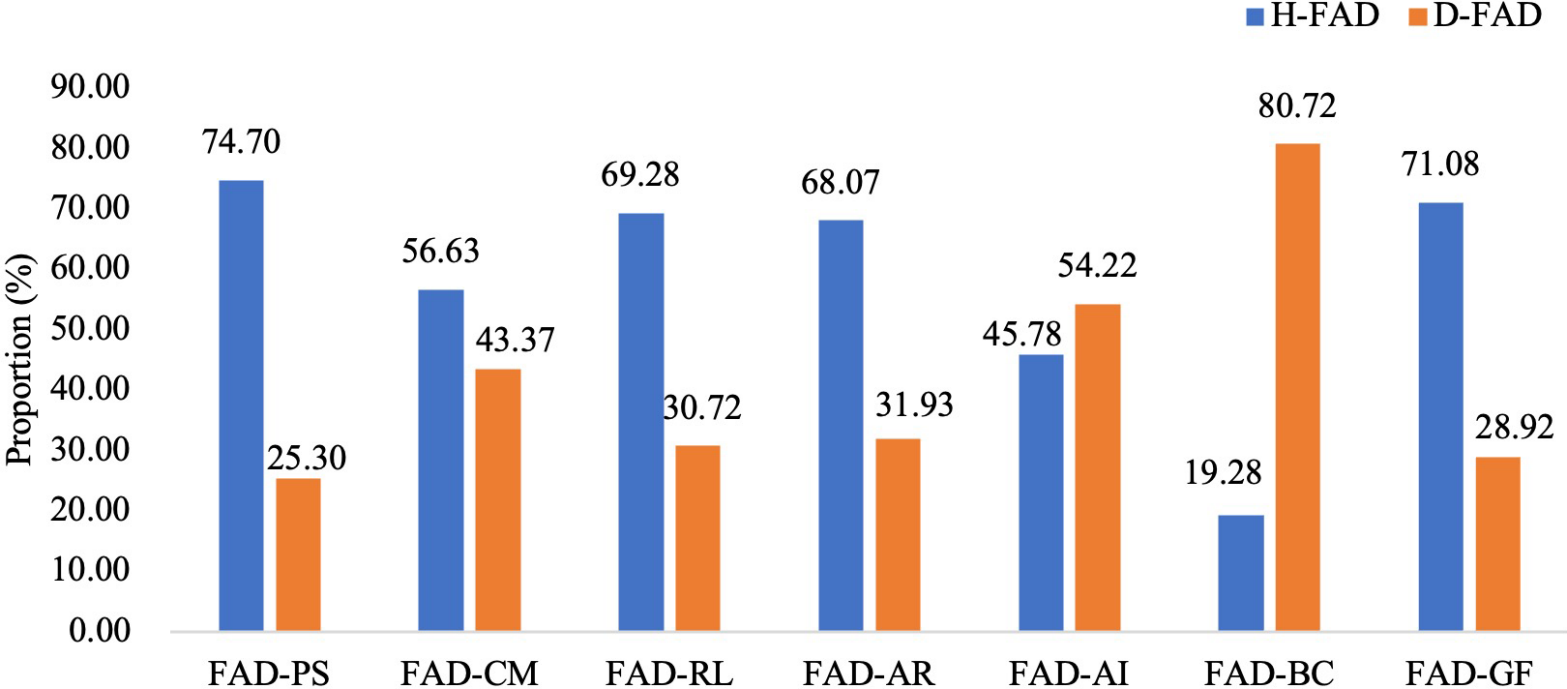
Proportion of partners with healthy and dysfunctional Family Assessment Device (FAD) subscales (%).

In males, the mean scores of FAD-PS, CM, RL, AR, and GF (all *p* < 0.01) were also below the cut-off scores. Mean scores of FAD-BC (*t =* 11.576, *p* < 0.001) were higher than the cut-off score, too. However, scores of FAD-AI showed no statistical difference with the cut-off score (*t =* −0.516, *p* = 0.607). Meanwhile, data on the proportion of partners with healthy and dysfunctional FAD subscales were presented in Figure 2. The FAD-BC ranks highest in dysfunctional rate and its proportion also prominently exceeded that of the healthy FAD-BC (80.72% vs. 19.28%).

### 3.3. Factors related to FAD-BC in participants

Differences in demographic and clinical characteristics between healthy and dysfunctional FAD-BC groups in participants were presented in Table 3. The results showed higher EPDS/PHQ-9 and GAD-7 scores in the dysfunctional FAD-BC groups compared to the healthy groups in participants (all *p* < 0.05).

**Table 3:** Differences in demographic and clinical characteristics between healthy and dysfunctional FAD-BC groups in participants.

Item	Pregnant females (*n* = 226)				Partners (*n* = 166)			
	H-FAD-BC (*n* = 36)	D-FAD-BC (*n* = 190)	*χ*^2^/*Z*/*t* value	*p* value	H-FAD-BC (*n* = 32)	D-FAD-BC (*n* = 134)	*χ*^2^/*Z* value	*p* value
Marital status, married (%)^a^	27 (75.0)	166 (87.4)	3.713	0.054	28 (87.5)	113 (84.3)	0.031	0.861
Occupational status, in employment (%)^a^	24 (66.7)	147 (77.4)	1.882	0.170	32 (100.0)	132 (98.5)	-	1.000
History of drinking^a^	2 (5.6)	10 (5.3)	<0.001	1.000	21 (65.6)	69 (51.5)	2.078	0.149
History of smoking^a^	2 (5.6)	9 (4.7)	<0.001	1.000	12 (37.5)	67 (50.0)	1.618	0.203
Planned pregnancy^a^	24 (66.7)	119 (62.6)	0.212	0.645	22 (68.8)	79 (59.0)	1.040	0.308
Subscale scores of FAD^b^	1.83 (0.22)	2.28 (0.33)	−9.594	**<0.001**	1.78 (0.22)	2.22 (0.33)	−8.837	**<0.001**
Total scores of EPDS^b^	5.06 ± 3.40	6.50 (5)	−2.795	**0.005**	–	–	–	–
Total scores of PHQ-9^b^	–	–	–	–	2 (4)	4 (6)	−3.145	**0.002**
Total scores of GAD-7^b^	2 (3)	3 (3)	−2.017	**0.044**	1 (4)	3 (5)	−2.654	**0.008**
Total scores of SSRS^c^	38.72 ± 6.10	37.92 ± 5.75	0.760	0.448	–	–	–	–
Total scores of Q-LES-Q-SF^b^	54.78 ± 7.76	53 (8)	−1.534	0.125	–	–	–	–

Bold values indicate statistical significance; Data are shown as mean ± standard deviation, median (interquartile range), or *n* (%) in different categories.

a Chi-square test.

b Mann-Whitney *U* test.

c Two independent samples *t*-test. *p* value: Differences in demographic and clinical characteristics between healthy and dysfunctional FAD-BC groups in pregnant females or partners.

FAD, Family Assessment Device; BC, Behavior Control; H-FAD, Healthy FAD; D-FAD, Dysfunctional FAD; EPDS, Edinburgh Postnatal Depression Scale; PHQ-9, Patient Health Questionnaire-9; GAD-7, Generalized Anxiety Disorder 7-item; SSRS, Social Support Rating Scale; Q-LES-Q-SF, Quality of Life Enjoyment and Satisfaction Questionnaire, Short Form.

Factors related to dysfunctional FAD-BC in participants were shown in Table 4. In pregnant females, the length of time living with a partner, total scores of EPDS, GAD-7, and Q-LES-Q-SF were all significantly correlated with the scores of dysfunctional FAD-BC (*r* = −0.165, 0.195, 0.148, and − 0.231, respectively; all *p* < 0.05). In male partners, the total scores of PHQ-9 and GAD-7 were both positively associated with the scores of dysfunctional FAD-BC (*r* = 0.275 and 0.298 respectively; both *p* < 0.01).

**Table 4:** Factors related to dysfunctional FAD-BC in participants.

Item	D-FAD-BC in pregnant females (*n* = 190)	D-FAD-BC in partners (*n* = 134)
Age^a^	−0.085	−0.022
BMI^a^	0.026	−0.005
Years of education^a^	0.008	−0.063
Length of time living with a partner^a^	−0.165^*^	−0.114
Gestational weeks^a^	−0.137	0.054
Total scores of EPDS^a^	**0.195** ^**,^ ^b^	–
Total scores of PHQ-9^a^	–	**0.275** ^**^
Total scores of GAD-7	0.148^*,^ ^b^	0.298^***,^ ^a^
Total scores of SSRS^a^	−0.020	–
Total scores of Q-LES-Q-SF^b^	−0.231^**^	–

Bold values indicate statistical significance; Data are shown as *r* coefficients.

a Spearman correlation analysis.

b Spearman partial correlation analysis. ^*^P_r_ < 0.05; ^**^P_r_ < 0.01; ^***^P_r_ < 0.001.

FAD, Family Assessment Device; BC, Behavior Control; D-FAD, Dysfunctional FAD; BMI, Body Mass Index; EPDS, Edinburgh Postnatal Depression Scale; PHQ-9, Patient Health Questionnaire-9; GAD-7, Generalized Anxiety Disorder 7-item; SSRS, Social Support Rating Scale; Q-LES-Q-SF, Quality of Life Enjoyment and Satisfaction Questionnaire, Short Form.

## 4. Discussion

Numerous studies have acknowledged the negative impact of family dysfunction on the general population or individuals with mental disorders, but the understanding of family functioning in early pregnancy remains unclear. Based on the established knowledge of the special family functioning problems, this study focused on the characteristics of family functioning in early pregnancy and associated factors with promoting a basic understanding of family functioning in pregnant women and their partners.

In this study, BC was the only dimension of family functioning that was dysfunctional in participants. In particular, the rate of dysfunctional BC was the highest and prominently exceeded that of healthy BC both in pregnant women and partners compared to other dimensions. According to the MMFF theory, family functioning of BC refers to family patterns that deal with family members’ behaviors to meet and express needs and motivations in risky situations or social environments, including “We do not know what to do when an emergency comes up” “If the rules are broken, we do not know what to expect” “There are rules about dealing with dangerous situations” and so on (Epstein et al., 1983). Findings in the present study suggest that pregnant families may face more adaptive behavior conflicts in various situations. Under the environment of social transition, Chinese traditional family values, obligation, and collectivism have been challenged by the rapid social transition in urbanization and industrialization, as well as the promotion of individualism (Xu and Xia, 2014). Therefore, family rules under the transition of the sociocultural environment might face more challenges, especially during pregnancy.

Improving the quality of family functioning are likely to promote mental health among family members. In the present study, early-pregnancy females and males with poor family functioning of BC showed more severe depressive and anxious symptoms. Available evidence supported the finding, as researchers revealed that family dysfunction is an accompaniment of an acute depressive episode commonly, and consistently impaired family functioning might influence the course and severity of depression (Gabor and Ivan, 1990). Family functioning was also important in predicting perinatal depression and related interventions (Huang et al., 2021). Within the biobehavioral family model (BBFM), the approach posits that family emotional climate, the quality of parent relations and parent–child relationship security, and biobehavioral reactivity can influence each other collectively in stress-related illness. It reveals that an insecure family environment and low-quality parent relations would generate implicit and explicit impacts on each individual in the family (Wood et al., 2008). BBFM values family in three aspects, including the intensity of emotional exchange, parent interaction patterns in support, understanding, and conflict. These perspectives reflect the quality of family functioning laterally and are tightly related to each family member’s performance in BC. Combining clinical and theoretical findings, family functioning plays a vital role in adjusting the individual’s mental health. It also underlined a critical aspect of environmental factors leading to mental illness and provided a potential pathway to promote females’ and partners’ mental health during the first trimester.

Additionally, dysfunctional BC was negatively associated with the time lived with a partner and the quality of life in females. It indicates that the longer females and their partners live together, the better the family functioning of BC might have. Traditionally, Chinese culture values the continuity of ethnicity and bloodline. Newly married couples are likely expected to have a pregnancy at an early stage after marriage (Yang et al., 2018). Even though many families and young people have been affected by the new age thinking in relationships and marriage by cross-cultural concepts and individualism, cohabitation has started emerging but remains marginalized in the Chinese population (Xu and Xia, 2014). Thus, before marriage and childbirth, parents during early pregnancy might spend less time adapting to the family environment and new responsibilities as first-time parents and make adjustments in BC. Besides, researchers also revealed that BC is largely independent of other FAD scales as it determines the vital pattern of the standard behavior in each family (Ridenour et al., 1999) and suggests that BC can distinguish clinical and nonclinical families (Miller et al., 1985). These findings reinforced the significance and uniqueness of BC in family functioning. Also, it offers a potential pathway for family education and mental health providers to help families in early pregnancy improve family mental health and the functioning of BC under various situations by intervening in BC independently and assisting parents during early pregnancy adapting to family environments.

In sum, the results of the present study highlighted that maintaining good family functioning in BC might be a challenge for women and partners during early pregnancy, which requires more clinical attention and health education. It also provided insight into improving family wellness and pinpointing guidelines to promote family functioning during early pregnancy.

## 5. Limitations

Although this study found some clinically significant results, it still had the following shortcomings. Firstly, it was a cross-sectional study that could not reveal the characteristics of changes in family functioning during pregnancy. Secondly, there might be some differences and correlations in family functioning between pregnant females and partners generally. For example, in the current study, the mean score of FAD-AI in females was significantly below the cut-off score while there was no statistical significance in partners. Considering some partners of pregnant women failed to participate in the present study, we could not examine the themes mentioned above in the form of maternal-partner pairs because this paired format may better facilitate the exploration of differences and correlations. Thirdly, the data collection from partners in this study was primarily conducted online, given the relative complexity of completing the SSRS and Q-LES-Q-SF scales that may require on-site instruction, so in this case, partners’ social support and quality of life were not assessed. Additionally, factors related to family functioning included in this paper were not comprehensive. In general, the maternal pressures and opinions are different from their partners, especially in caring baby, and these may also further impact the family function, which was not explored in this study and needed to be included in the future.

## 6. Conclusion

Early pregnancy is a vulnerable period of dysfunctional family functioning. The current study revealed that the dysfunctional family functioning of BC was most prominent both in females and partners in early pregnancy. Length of time living with a partner, depressive and anxious symptoms, and quality of life were associated with dysfunctional family functioning of BC in this period. The findings in this research highlighted the need for public and clinical awareness of family functioning during early pregnancy and underlined a possible guideline for family education for this period.

## Data availability statement

The original contributions presented in the study are included in the article/supplementary material, further inquiries can be directed to the corresponding author.

## Ethics statement

The studies involving human participants were reviewed and approved by The Second Xiangya Hospital of Central South University. The patients/participants provided their written informed consent to participate in this study.

## Author contributions

LL, YZ, BL, and XQ contributed to the study’s conception and design. XQ, SX, MM, XF, XN, JL, YJ, LZ, and BL are responsible for participant recruitment and data collection. XQ and WZ did the data analysis and wrote the first draft of the manuscript and revisions. BL, XQ, YZ, and HL contributed substantial revisions to the manuscript. All authors contributed to the article and approved the submitted version.

## Funding

The work has been supported by The National Natural Science Foundation of China (82001437 and 82171518); and the Key Program of Hunan Health Commission (202205033887). The funding sources had no role in the study design, data collection and analysis, interpretation of the data, preparation and approval of the manuscript, and decision to submit the manuscript for publication.

## Conflict of interest

The authors declare that the research was conducted in the absence of any commercial or financial relationships that could be construed as a potential conflict of interest.

## Publisher’s note

All claims expressed in this article are solely those of the authors and do not necessarily represent those of their affiliated organizations, or those of the publisher, the editors and the reviewers. Any product that may be evaluated in this article, or claim that may be made by its manufacturer, is not guaranteed or endorsed by the publisher.
